# Lactoferricin B reverses cisplatin resistance in head and neck squamous cell carcinoma cells through targeting PD‐L1

**DOI:** 10.1002/cam4.1529

**Published:** 2018-05-15

**Authors:** Pei Zhang, Jinzhong Liu, Wenlu Li, Shanshan Li, Xinguang Han

**Affiliations:** ^1^ Department of Stomatology The First Affiliated Hospital of Zhengzhou University Zhengzhou China; ^2^ Key Laboratory of Tumor Pathology Department of Pathology The First Affiliated Hospital of Zhengzhou University Zhengzhou China

**Keywords:** chemoresistance, head and neck squamous cell carcinoma, lactoferricin B, programmed *death‐ligand* 1

## Abstract

Head and neck squamous cell carcinoma (HNSCC) ranks among the top most common cancers with a poor prognosis. The mechanism of chemoresistance is still not well known. This study is to investigate the programmed *death‐ligand* 1 (PD‐L1) expression in HNSCC, and test the effect of lactoferricin B (LfcinB) on chemoresistance and its mechanism. We analyzed 510 HNSCC patients in TCGA database and investigated how CD274 expression was related to patient prognosis. PD‐L1 was verified from HNSCC samples at local hospital with immunohistochemistry. PD‐L1 expression in the acquired cisplatin‐resistant HNSCC cells was examined by PCR and WB in order to test PD‐L1‐induced chemoresistance. LfcinB inoculation in cisplatin‐resistant HNSCC cells and in the nude mice was introduced to test the effect of LfcinB on targeting cisplatin resistance and its mechanism. High CD274 mRNA (>125 FPKM) from TCGA database had a significantly reduced 5‐year survival rate, and a lower 5‐year survival rate in the chemotherapy and radiotherapy‐treated patients (*P *< .05). PD‐L1 overexpression was further supported from analysis of 40 HNSCC specimens. PD‐L1 and IL‐6 in the established cisplatin‐resistant HNSCC cells were shown significantly higher (*P <* .05). IL‐6 and PD‐L1 expression were partially inhibited by the anti‐IL‐6/STAT3 antibody. LfcinB displayed a direct cytotoxic effect on cisplatin‐resistant HNSCC cells and HNSCC xenografts of cisplatin‐resistant cells in the nude mice displayed significant reduction in tumor volume after LfcinB injection (*P *<* *.05). Besides, the increase of IL‐6 and PD‐L1 in cisplatin‐resistant HNSCC cells was abolished in vitro by LfcinB (*P *< .05). PD‐L1 expression in HNSCC cells correlates with poor prognosis and chemoresistance, and LfcinB might provide therapeutic potential in HNSCC patients through modulating IL‐6 and PD‐L1.

## 1 INTRODUCTION

Head and neck cancer ranks among the top most common cancers worldwide and head and neck squamous cell carcinoma (HNSCC) accounts for nearly 90% of head and neck cancer with a number of 644 000 cases are diagnosed worldwide each year.^1^ Despite the advances in cancer diagnosis and therapy, including surgery, radiotherapy, and chemotherapy, HNSCC ranks among the tumor types with a poor prognosis and the 5‐year survival rate remains about 50%,^2^, ^3^ partly because the chemoresistance limits its efficiency. The previous work demonstrated that the increased expression of interleukin 6 (IL‐6) is associated with poor prognosis and cisplatin‐acquired chemoresistance of HNSCC^4^ while the mechanism of chemoresistance is still not clear. Immune suppression in the tumor microenvironment may be introduced and maintained through programmed cell death protein (PD‐1)/programmed death*‐*ligand 1 (PD‐L1) suppressing the functions of T cells from a broad range of cancer types, including HNSCC.^5^, ^6^ Few studies in myeloma cells concerning the markers between immune suppression and drug resistance were reported.^7^, ^8^

Lactoferricin B (LfcinB), cationic antimicrobial peptides, can selectively kill many types of tumor cells through targeting the cell membrane by electrostatic interactions with anionic molecules at the surfaces of cancer cells independent of multidrug‐resistance mechanism.^9^, ^10^ As LfcinB was tested for the cytotoxic effect against the oral cavity squamous cell carcinoma and HNSCC cells,^11^, ^12^ LfcinB may offer hope to break down the chemoresistance of HNSCC while further investigations focused on mechanism are needed.

The aim of this study was to evaluate whether PD‐L1 overexpression might have a prognostic value in HNSCC and influence the cisplatin resistance of HNSCC. We analyzed clinical data of 510 HNSCC patients in the cancer genomic atlas (TCGA) database and investigated how CD274 (gene type of PD‐L1) expression was related to patient prognosis. In order to examine PD‐L1 induced cisplatin resistance, we furthermore tested cisplatin sensitivity and PD‐L1 expression in the acquired cisplatin‐resistant HNSCC cell lines. Furthermore, in vitro inoculation of LfcinB in cisplatin‐resistant HNSCC cells and in vivo local injection of LfcinB into the implanted HNSCC tumor in the nude mice were introduced to test the effect of LfcinB on targeting cisplatin resistance and its mechanism.

## 2 MATERIALS AND METHODS

### 2.1 Clinical data and PD‐L1 (CD274) expression analysis

Clinical and RNA‐seq data from 510 HNSCC patients were obtained from TCGA project (https://cancergenome.nih.gov). All patients, diagnosed and treated during 1997‐2017, were followed until October 9, 2017. Clinicopathological characteristics, including age, gender, tumor location, histological grade, TNM stage, treatment, HPV status, smoking and alcohol history, and overall survival information were collected. For detailed tumor sample acquisition, see reference 13. Briefly, tumor specimens were collected at the time of surgical resection. The patients had received no chemo‐or radiotherapy prior for their disease. Cases were staged according to the American Joint Committee on Cancer (AJCC), Seventh Edition.^14^ The mRNA expression profiles were estimated by normalizing raw counts of mapped RNA‐sequences reads to human reference genes, and CD274 mRNA level measured as fragments per kilobase per million mapped reads (FPKM). Patients without complete follow‐up data, complete RNAseq information or who died within 2 months were excluded, and finally, 510 patients were included in the study.

HNSCC tissues samples (n = 40) were obtained for analysis in 2015 from patients undergoing biopsy in Department of Stomatology and Department of Pathology at the First Affiliated Hospital of Zhengzhou University. No patients received any treatment before the biopsy, including radiotherapy and chemotherapy. The diagnosis of HNSCC was confirmed by at least an experienced pathologist. The resected tissues were fixed in 10% buffered formaldehyde solution for pathologic diagnosis and immunohistochemistry analysis.^15^

### 2.2 HNSCC Cell lines and establishing the cisplatin‐resistant HNSCC cell lines

Human HNSCC lines, CAL27, and Detroit‐562 (purchased from the American Type Culture Collection, ATCC) were used in the study and were cultured under standard condition as described.^4^ The 2 primary cisplatin sensitive HNSCC cell lines, oral tongue squamous cell carcinoma, CAL27, and pharyngeal squamous carcinoma, Detroit‐562 were cultured to acquire cisplatin resistance. Cells were treated with gradually increasing concentrations of cisplatin (Qilu Pharmaceutical Co., Ltd, Jinan, China) ranging from 3 to 10 μmol/L at weekly intervals for 8 months to establish cisplatin‐resistant CAL27 (CAL27cis) and Detroit‐562 (Detroit‐562cis) cell lines. The establishment of cisplatin‐resistant cells was selected by exposure to sequential cycles of cisplatin for 8 months, which mimics the way the drug is used in the clinic.^4^, ^16^ The resistance was unaltered after culturing the cisplatin‐resistant cells in the drug‐free medium for 2 months. CAL27, Detroit‐562, CAL27cis, and Detroit‐562cis were inoculated with LfcinB for test of drug resistance and the expression of PD‐L1 and IL‐6. LfcinB (FKCRRWQWRMKKLGAPSITCVRRAF), 25‐AA peptide fragment, was synthesized by GL Biochem (Shanghai, China) via a stepwise solid phase methodology. The peptide was purified by a Sephadex gel column and HPLC, and the homogeneity of the purified peptide was greater than 98%.

### 2.3 In vivo tumor xenograft experiment

To analyze the direct effect of LfcinB on cisplatin‐resistant squamous carcinoma cells, the xenograft experiment of in vivo growth of CAL27cis or Detroit‐562cis in nude mice was carried out. CAL27cis or Detroit‐562cis cells (1 × 10^6^ per mice) were implanted subcutaneously into BALB/c mice. When the tumors reached approximately 6 mm in diameter, a dosage of 0.75 mg LfcinB in 100 μL saline (Group LfcinB) or 100 μL saline (Group Control) per day was intratumorally injected into nude mice for 3 days. Tumor growth and tumor size were monitored using caliper every 2 days.

### 2.4 MTT assay

The in vitro cytotoxic effects of cisplatin or LfcinB on CAL27cis and Detroit‐562cis were tested in a standard MTT assay. For drug IC50 detection, CAL27cis or Detroit‐562cis in 96‐well microtitre plates (Corning, Bibby Sterilin Ltd, Staffordshire, U.K.) were treated with different dosages of cisplatin or LfcinB. After the incubation period of 24 hours, a standard MTT assay was performed as described in detail previously.^4^ IC50 was calculated using GraphPad Prism 6.0 (GraphPad Software, Inc., La Jolla, CA, USA). The cytotoxic effect of cisplatin or LfcinB was expressed as the relative viability (% of control) and was calculated as follows: Relative viability = (experimental absorbance − background absorbance)/(absorbance of untreated controls‐background absorbance) × 100%.

### 2.5 Immunohistochemistry and immunohistochemical analysis

Sections of formalin‐fixed paraffin‐embedded tissues were prepared and stained with H&E. Immunohistochemistry (IHC) was performed on 4 μm tissue sections using a Ventana Bench Mark XT Autostainer (Ventana Medical Systems, Tucson, USA). PD‐L1 expression on tumor cells was analyzed by applying a modification of a previously used approach.^15^ Primary antibody against PD‐L1 (dilution 1:100; NBP1‐76769; Novus Biologicals, Littleton, USA) was used. PD‐L1 staining was evaluated by 2 independent pathologists with frequency (from 0% to 100%) and the intensity (negative or trace, weak, moderate, intense) per slide. PD‐L1 positivity was defined as PD‐L1 expression in at least 5% of tumor cells ranging from moderate to intense based on IHC staining by examining 5 representative high‐power fields under 200× magnifications.

### 2.6 Quantitative real‐time reverse transcriptase polymerase chain reaction (qRT‐PCR)

After RNA isolation of CAL27cis and Detroit‐562cis, complementary DNA (cDNA) was synthesized by RT‐RTCK‐05 kit (Eurogentec, Berlin, Germany) and stored at −20°C. A standard real‐time PCR reaction with SYBR Green Real Master Mix (Eppendorf, Hamburg, Germany) was performed in duplicates using Mx3005p (Agilent Technologies) under the following conditions: 95°C for 2 minutes followed by 40 cycles of 95°C for 20 seconds, 60°C for 1 minutes and 68°C for 30 seconds. The primers used were: PD‐L1 forward, 5′‐ TCAATGCCCCATACAACAAA ‐3′ and reverse, 5′‐ TGCTTGTCCAGATGACTTCG ‐3′; IL‐6 forward, 5′‐GCAGAAAAAGGCAAAGAA TC‐3′ and reverse, 5′‐CTACATTTGCCGAAGAGC‐3′; GAPDH forward, 5′‐GGATTTGGTCGTA TTGGG‐3′, reverse, 5′‐GGAAGATGGTGATGGGA TT‐3′ (all designed by Sigma‐Aldrich). Expression data were normalized to the housekeeping gene GAPDH. The relative expression levels of the genes of interest were calculated using the 2^−ΔΔ*Ct*^ method.

### 2.7 Western blotting

HNSCC lines cells were harvested and lysed in CelLytic M Cell Lysis reagent (Sigma‐Aldrich, St. Louis, MO, USA) with protease and phosphatase inhibitor cocktails (Pierce Biotechnology, Rockford, USA). Protein concentrations were determined (Bio‐Rad, Munich, Germany). Standard Western blotting (WB) assay was used to analyze protein expression, as described previously. Briefly, immunostaining was detected with primary antibody to PD‐L1 (rabbit polyclonal, dilution 1:1000; NBP1‐76769; Novus Biologicals, Littleton, USA), anti‐β‐tubulin (mouse monoclonal, 1:1000; Abcam, Cambridge, UK) and anti‐rabbit IgG (1:10 000; Sigma‐Aldrich) or mouse IgG (1:10 000; Dako, Glostrup, Denmark) antibodies The immunoreactive signals were visualized by scanning densitometry with ChemiDoc^™^ Touch Imaging System.

### 2.8 Enzyme‐linked immunosorbent assay (ELISA)

CAL27, Detroit‐562, CAL27cis, and Detroit‐562cis cells were seeded in duplicates in 96‐well plate at a density of 5 × 10^3^ cells per well and cultured in 200 μL medium with 10% serum. After allowing cells to attach overnight, the new medium was added and then supernatant and cells were collected at 6 and 24 hours, respectively. The supernatant and cells were harvested and stored frozen (−70°C) for ELISA, WB and qRT‐PCR. The IL‐6 concentration was determined in quadruplicates by Human IL‐6 ELISA kit (R&D Systems, Minneapolis, USA).

### 2.9 Statistics

Statistical analysis was performed using GraphPad prism 6.0 (San Diego, California, USA). The survival distributions were compared with the log‐rank test (Kaplan‐Meier method). Deaths from any cause were defined as events. The patients were censored at loss to follow‐up, defined as the last date of contact or at 5 years after diagnosis. Normally distributed data were shown as mean ± SD, and group differences were analyzed using Student’s *t* test. A *P*‐value of less than .05 was considered statistically significant.

## 3 RESULTS

### 3.1 High expressions of CD274 (PD‐L1) in the tumor predicts poor prognosis

**Figure 1:**
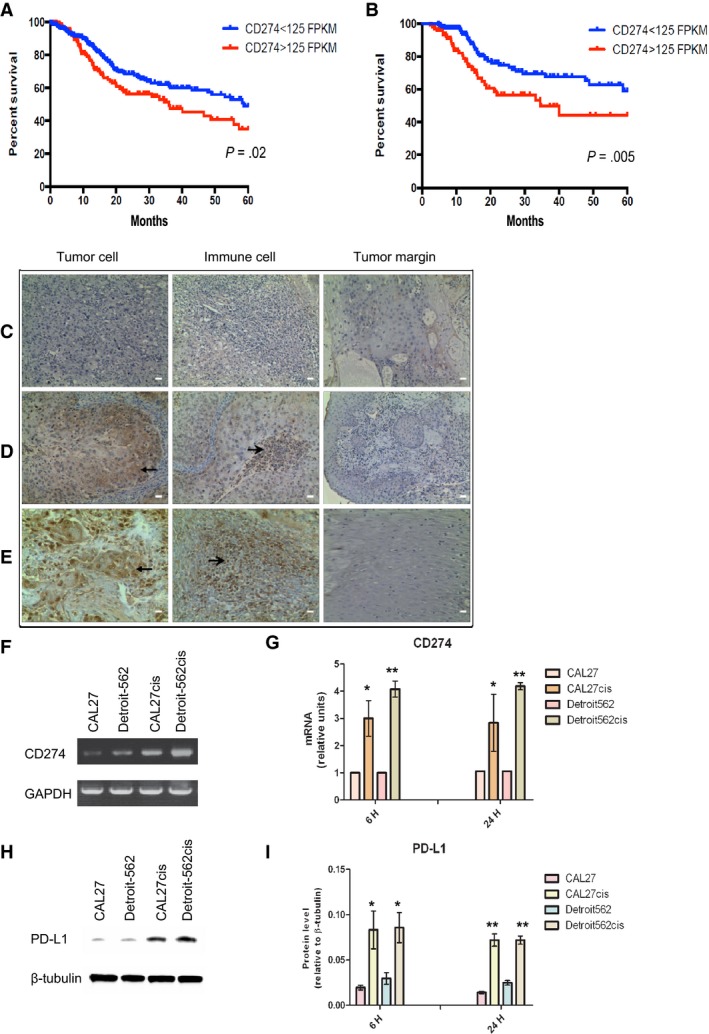
CD274 (PD‐L1) expression in the HNSCC patients from the TCGA database (A, B), HNSCC tissue samples (C, D, E) and the HNSCC cells (F, G, H, I). CD274 expression with survival and its relation with therapy in the HNSCC patients from the TCGA database (A, B): High CD274mRNA levels (>125 FPKM) predicted poor prognosis in all patients (*P *=* *.02) (A) and in chemotherapy and radiotherapy treated patients (*P* = .005) (B). CD274 mRNA levels measured as fragments per kilobase per million mapped reads (FPKM). Immunostaining of PD‐L1 obtained from HNSCC tumor cells, immune cells and tumor margin tissues in HNSCC tissue samples (magnification ×200, scale bars 50 μm) (C, D, E): low tumor staining (C); moderate tumor staining (D); high tumor staining (E). Brown staining stands for the PD‐L1 positive cells as indicated by black arrows. Expression of CD274 gene and PD‐L1 protein in the established cisplatin‐resistant HNSCC cells and cisplatin sensitive cells by qRT‐PCR and WB (F, H): CD274 (PD‐L1) expressed in cisplatin‐resistant cells, CAL27cis and Detroit‐562cis, were shown significantly higher than the cisplatin‐sensitive cell lines, CAL27 and Detroit‐562 (G, I). Data are shown as mean ± SD of 3 independent triplicate cultures (G, I). *P < .05 and **P < .01 for cisplatin‐resistant cells compared with the results of cisplatin‐sensitive cells

We firstly analyzed the clinical data of 510 HNSCC patients and the expression CD274 (PD‐L1) of these patients in TCGA database. Clinical and histological characteristics HNSCC patients in TCGA database were collected and summarized in Table 1, Figure 1A,B. The positive percentage of CD274 gene of 510 patients in TCGA database was 100% (510/510). To assess whether PD‐L1 in HNSCC tumors were biologically active, PD‐L1 positivity was found 92.5% (37/40) of HNSCC specimens from IHC analysis (Figure 1C‐E).

**Table 1:** Clinical and histological characteristics HNSCC patients in TCGA database

Characteristic	N	%
Gender		
Male	375	74
Female	135	26
Age (y)		
≤30	8	2
31‐40	13	3
41‐50	73	14
51‐60	157	31
61‐70	156	30
71‐80	79	15
81‐90	24	5
Tumor sites		
Oral cavity	308	61
Oropharynx	78	15
Larynx	114	22
Hypopharynx	10	2
Tumor pathological stage		
I	26	5
II	69	14
III	81	16
IV	260	51
Unknown	74	14
Tumor histological grade		
G1	62	12
G2	296	58
G3	123	24
G4	7	1
GX/Unknown	22	5
Smoking history		
Smoker	384	75
Non‐smoker	114	23
Unknown	12	2
Alcohol history		
Alcohol consumption	339	67
No alcohol consumption	160	31
Unknown	11	2
HPV status		
Positive	39	7
Negative	80	16
Unknown	391	77
Chemotherapy		
Cisplatin (carboplatin/oxaliplatin)	88 (56)	17 (11)
Other drugs	19	4
No chemotherapy	347	68
Radiotherapy		
Yes	302	59
No	208	41

To evaluate whether PD‐L1 overexpression in the tumor might have a prognostic value in HNSCC, dividing patients in high (>125 FPKM) and low (<125 FPKM) CD274‐expressed levels from TCGA database revealed that the high CD274 mRNA expressing group had a significantly reduced 5‐year survival rate (Figure 1A). Further investigation of patients after treatment of both chemotherapy and radiotherapy showed those with high levels of CD274 mRNA expression tended to have a lower 5‐year survival rate (Figure 1B). Thus, an increased CD274 gene expression in the HNSCC tumors was related to poor prognosis and presumably also to chemoresistance.

### 3.2 PD‐L1 and IL‐6 in the cisplatin‐resistant HNSCC cells

The IC50 values for cisplatin‐treated cells, CAL27cis and Detroit‐562cis cells, were 3 times higher than IC50 in the parental cells (CAL27 and Detroit‐562 cells) (Table 2). To examine whether PD‐L1 is involved in cisplatin resistance in HNSCC, expression of CD274 and PD‐L1 in the established cisplatin‐resistant HNSCC cells, CAL27cis and Detroit‐562cis, were shown significantly higher than the cisplatin sensitive cell lines by qRT‐PCR and WB (Figure 1F‐I).

**Table 2:** Characterization of HNSCC cell lines

	IC50 for cisplatin (μmol/L)	IC50 for lactoferricin B (μmol/L)
CAL27	2.9 ± 1.1	8.1 ± 0.8
CAL27cis	8.6 ± 1.0^a^	10.2 ± 1.1^a^
Detroit‐562	3.2 ± 0.4	10.3 ± 1.1
Detroit‐562cis	10.3 ± 3.6^a^	12.7 ± 1.1^a^

Data are shown as mean ± SEM, n = 3. Cells were plated into 96‐well plates (5 × 10^3^ cells/well) and cultured at 37°C. Absorbance was examined by MTT array. DT was calculated using the following equation: DT(h) = (log2 × *t*)(logA2−logA1), where *t* = time in culture (h), At = final absorbance, A0 = original absorbance.

Represents statistic significance compared to parent cells (*P *= .001 and .001).

a Represents no statistic significance compared to parent cell lines (*P = *.22 and .25).

**Figure 2:**
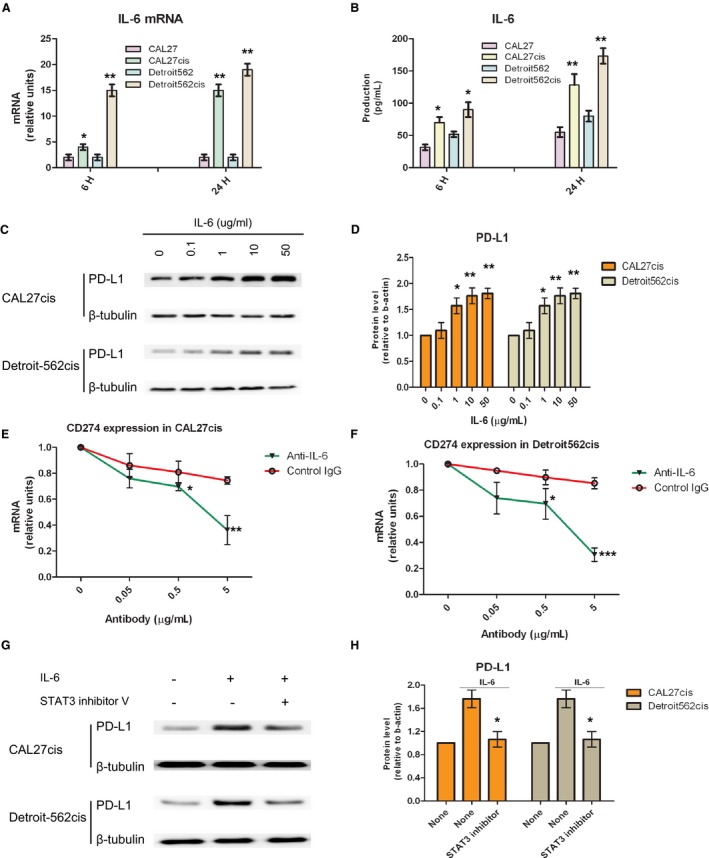
Upregulated expression of PD‐L1 in cisplatin‐resistant cells through IL‐6/STAT3. The cisplatin resistant CAL27cis and Detroit‐562cis cells had increased IL‐6 mRNA and IL‐6 protein production compared to parental cells by qRT‐PCR and ELISA (A, B). CAL27cis and Detroit‐562cis were cultured with IL‐6 (10 ng/mL, human recombinant IL‐6, R&D Systems, Minneapolis, USA) for 48 h (C). An anti‐human IL‐6 polyclonal antibody (R&D Systems, Minneapolis, USA) was used to neutralize the biological activities of IL‐6 (E, F). To inhibit signal transduction, CAL27cis and Detroit‐562cis cells were pre‐incubated for 1 h with STAT3 inhibitor V (Sigma‐Aldrich, Germany, 500 nmol/L), and then incubated with recombinant human IL‐6 (10 ng/mL) for 24 h before analysis. Data are shown as mean ± SD of 3 independent triplicate cultures (A, B, D, E, F, H). *P < .05, **P < .01 and ***P < .001, compared with the results of control without IL‐6 (D), control IgG (E, F) and with IL‐6 alone (H)

The previous study showed the acquired cisplatin‐resistant HNSCC cells markedly increased the cisplatin‐induced IL‐6 expression,^4^ which was verified in this study (Figure 2A,B). The cisplatin‐resistant CAL27cis and Detroit‐562cis cells increased cellular IL‐6 mRNA and supernatant IL‐6 protein production compared to parental cells (CAL27 and Detroit‐562) by qRT‐PCR and ELISA (Figure 2A,B).

In this study, we also examined that IL‐6 could enhance PD‐L1 expression in the cisplatin‐resistant CAL27cis and Detroit‐562cis cells, and this enhancement was dose‐dependent (Figure 2C,D). Furthermore, IL‐6 mediated CD274 expression through IL‐6 pathway was indicated. This IL‐6‐inducing effect in the established cisplatin‐resistant cells was partially inhibited by an antagonistic antibody to IL‐6/STAT3 (Figure 2E‐H). As expected, the IL‐6‐induced PD‐L1 expression in cisplatin‐resistant CAL27cis and Detroit‐562cis cells was down‐regulated by inhibiting molecule involved in the IL‐6 signal cascade, STAT3.

### 3.3 Effect of LfcinB reducing cisplatin resistance in cisplatin‐resistant HNSCC cells in vitro and in vivo

**Figure 3:**
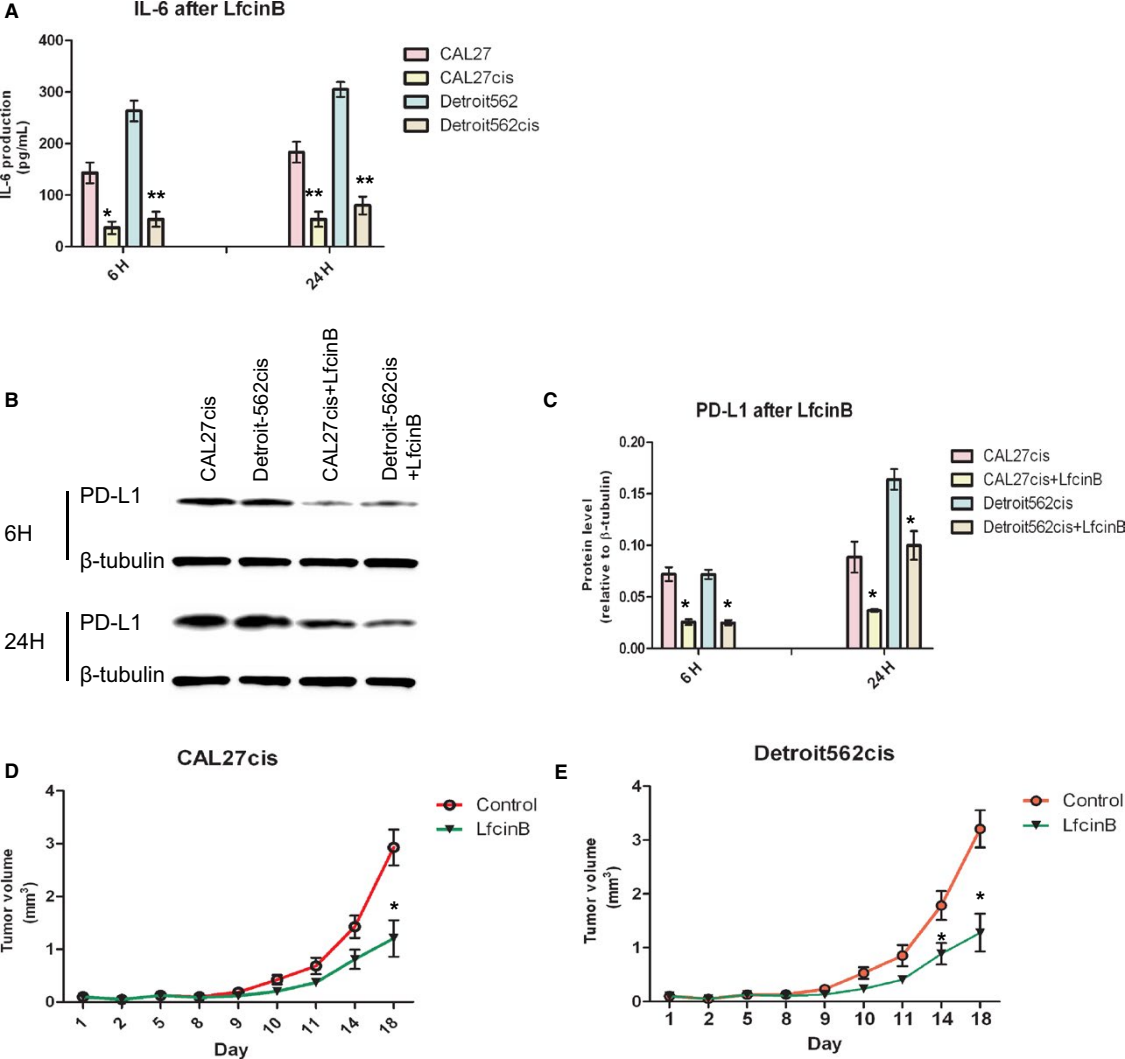
LfcinB reduced IL‐6‐dependent PD‐L1 expression in cisplatin‐resistant HNSCC cells (A‐C) and inhibited the xenograft growth from CAL27cis and Detroit‐562cis (D, E). The markedly decreased IL‐6 (A) by ELISA and PD‐L1 by WB (B, C) in the acquired cisplatin‐resistant cells were detected. In figure A, C and D, data are shown as mean ± SD of 3 independent triplicate cultures. *P < .05 and **P < .01, compared with the results of control without IL‐6 (A, C). To investigate the effects of LfcinB on in vivo growth of CAL27cis and Detroit‐562cis, we treated nude mice carrying CAL27cis (D) and Detroit‐562cis (E) xenografts with once‐daily injection of 0.75 mg LfcinB (n = 5). Tumor growth of HNSCC xenografts displayed significant reduction after 3 days of LfcinB treatment, compared to control with saline (*P < .05 for LfcinB)

IC50 values of cisplatin in the established cisplatin‐resistant HNSCC cells (CAL27cis and Detroit‐562cis cells) were significant higher than those in the parental cells (CAL27 and Detroit‐562 cells). LfcinB displayed a direct cytotoxic effect on both cisplatin‐resistant HNSCC cells and primary cisplatin sensitive HNSCC cells with no significance in IC_50_ values (Table 2), indicating LfcinB might overcome the established cisplatin resistance. The increase of IL‐6 and PD‐L1 in the cisplatin‐resistant HNSCC cells, which were shown to be involved in cisplatin resistance, was abolished by LfcinB with PCR and WB (Figure 3A‐C).

In the mice carrying growth of cisplatin‐resistant CAL27cis or Detroit‐562cis, HNSCC xenografts displayed a significant reduction in tumor volume after LfcinB injection (n = 5) for 3 days compared with the control animals (n = 5) (Figure 3D,E).

## 4 DISCUSSION

PD‐1/PD‐L1 mediated immune suppression in T‐cell immune suppression and the potential for immunotherapy via blocking PD‐L1/PD‐1 was highlighted in HNSCC.^5^, ^6^ Our analysis of PD‐L1 positivity from TCGA cohort and HNSCC specimens from the local hospital was above 90%, which is in agreement with the reported data (70%‐90%) in HNSCC,^17^, ^18^, ^19^ while the positivity from HNSCC was shown much higher than melanoma, and the other malignancies.^20^, ^21^ Our result indicates PD‐L1/PD‐1 checkpoint blockade has provided a grounded indication of anti‐tumor response of HNSCC treatment, where blocking the PD‐1/PD‐L1 axis might theoretically produce dramatic responses in HNSCC patients.^21^

It is well documented that PD‐L1/PD‐1 axis could suppress T‐cell function and maintain the immune suppression in tumor microenvironment.^5^, ^6^ Our clinical data of CD274 expression from the TCGA database indicates that the high CD274 (PD‐L1) expression in HNSCC (1) predicts a poor prognosis,^22^, ^23^ and (2) correlates with the poor reaction of chemotherapy and radiotherapy in HNSCC patients. Our result from the up‐regulated PD‐L1 in cisplatin‐resistant HNSCC cells provided evidences that PD‐L1 in tumor cells is associated with the enhancing aggressive characteristics of HNSCC cells, including resistance to chemotherapeutic drug. This poor reaction to chemotherapy was supported by the evidence that the PD‐L1/PD‐1 immune checkpoint might confer tumor cell chemoresistance, and blockade of PD‐L1/PD‐1 limited chemoresistance of malignancy.^24^ PD‐L1 molecular can be found expressed in both tumor cells and tumor‐infiltrating immune cells in the tumor. From an assessment of PD‐L1 in tumor cells and immune cells in HNSCC, the high PD‐L1 expression in immune cells instead of tumor cells is a favorable prognostic factor for HNSCC resection.^25^ PD‐L1 expression in HNSCC xenografts and its immunological mechanism is going to be investigated in the further studies. Besides, as we did not find the positive associations between PD‐L1 and the most common multidrug‐resistant (MDR) genes, including ABCC1 and ABCG2 from TCGA project (data not shown), PD‐L1‐related MDR genes needs to be further investigated.

IL‐6 from bone marrow stromal cells can augment myeloma cell growth, survival and drug resistance through up‐regulation of PD‐L1.^26^ The previous study showed the acquired cisplatin‐resistant cells markedly increased cisplatin‐induced IL‐6 expression, and the high tumorous IL‐6 mRNA expression had a significantly reduced 5‐year survival.^3^, ^4^ Its mechanism is assumed to relate with inhibition of tumorous apoptosis and induction of epithelial to mesenchymal transition by IL‐6, both of which increase drug resistance.^27^, ^28^, ^29^ Based on the established cisplatin‐resistant HNSCC cell lines, we found that the cisplatin resistance in HNSCC cells was related with up‐regulated IL‐6 and PD‐L1. Up‐regulation of CD274 (PD‐L1) is partly mediated through IL‐6/STAT3 pathway. The relation between IL‐6 and PD‐L1 is further supported by the inhibitive effect of LfcinB in the cisplatin‐resistant HNSCC cells. The modulation between PD‐L1 and IL‐6/STAT3 is still unclear. One possibility of the modulation may be mediated through the damage‐associated molecular, high mobility group box‐1 protein (HMGB1). HMGB1 was reported to stimulate IL‐6 secretion^30^ and change the tumor‐environment through PD‐L1.^31^

LfcinB can selectively kill many types of tumor cells through targeting the cell membrane and conquering multidrug‐resistance, including HNSCC cells.^9^, ^10^, ^11^ Our results indicated that one possible mechanism of LfcinB is to break down the drug resistance of tumor cells through down‐regulation of IL‐6 and PD‐L1. In vitro inoculation and in vivo local injection of LfcinB may reverse chemoresistance in HNSCC through PD‐L1. NK cells and macrophage in nude mice^32^ might infiltrate into the xenograft tumors, causing up‐regulation of IL‐6 and PD‐L1 in tumor microenvironment.^33^, ^34^ The expression of IL‐6/STAT3 and PD‐L1 of the HNSCC xenografts and its immunological mechanism after LfcinB injection is worthwhile for investigation in the further studies.

In conclusion our result showed that PD‐L1 (CD274) expression in tumor cells is associated with chemoresistance and the poor prognosis of HNSCC through IL‐6. LfcinB may have therapeutic potential in HNSCC patients through modulating IL‐6 and PD‐L1.

## CONFLICT OF INTERESTS

The authors declare no conflict of interests. None of the authors have any relevant financial relationship(s) with a commercial interest. The manuscript has not been related with any previous communication to a society or meeting.

## ETHICS

The Ethics Committee on Research of the First Affiliated Hospital of Zhengzhou University previously approved this study. All patients gave informed consent in accordance with institutional guidelines and continued ongoing medical supervision and assistance under the Helsinki Declaration.

Male BALB/c athymic nu/nu mice (Keli China Experimental Animal Center, Beijing, China) from Henan Experimental Animal Center (Zhengzhou, China) aged 6‐8 weeks, were used for the experiments within a temperature‐controlled environment with 12‐hours light/dark cycle, standard chow and water available ad libitum. Experimental protocols were approved by the animal ethics committee of Zhengzhou University (no reference number) and were carried out according to the institutional guidelines and reported in accordance with the ARRIVE guidelines.^35^
